# Increased Beat-to-Beat Variability of T-Wave Heterogeneity Measured From Standard 12-Lead Electrocardiogram Is Associated With Sudden Cardiac Death: A Case–Control Study

**DOI:** 10.3389/fphys.2020.01045

**Published:** 2020-08-25

**Authors:** Jenni J. Hekkanen, Tuomas V. Kenttä, Mira Anette E. Haukilahti, Janne T. Rahola, Lauri Holmström, Juha Vähätalo, Mikko P. Tulppo, Antti M. Kiviniemi, Lasse Pakanen, Olavi H. Ukkola, M. Juhani Junttila, Heikki V. Huikuri, Juha S. Perkiömäki

**Affiliations:** ^1^Research Unit of Internal Medicine, Medical Research Center Oulu, Oulu University Hospital, University of Oulu, Oulu, Finland; ^2^Forensic Medicine Unit, Finnish Institute for Health and Welfare, Oulu, Finland; ^3^Research Unit of Internal Medicine, Department of Forensic Medicine, Medical Research Center Oulu, University of Oulu, Oulu, Finland

**Keywords:** electrocardiography, T-wave, T-wave morphology, repolarization, sudden cardiac death, ventricular arrhythmias

## Abstract

**Introduction:**

The prognostic significance of beat-to-beat variability of spatial heterogeneity of repolarization measured from standard 12-lead ECG is not well-understood.

**Methods:**

We measured the short-term variability of repolarization parameters, such as T-wave heterogeneity in leads V4–V6 (TWH) and QT interval (QT), from five consecutive beats of previously recorded standard 12-lead ECG in 200 victims of unexpected sudden cardiac death (SCD) confirmed to be due to complicated atherosclerotic coronary artery disease (CAD) in medico-legal autopsy and 200 age- and sex-matched controls with angiographically confirmed CAD. The short-term variability of repolarization heterogeneity was defined as the standard deviation (SD) of the measured repolarization parameters. All ECGs were in sinus rhythm, and no premature ventricular contractions were included in the measured segment.

**Results:**

TWH-SD and QT-SD were significantly higher in SCD victims than in subjects with CAD (6.9 ± 5.6 μV vs. 3.8 ± 2.6 μV, *p* = 1.8E-11; 8.3 ± 13.1 ms vs. 3.8 ± 7.1 ms, *p* = 0.00003, respectively). After adjusting in the multivariate clinical model with factors, such as diabetes, RR interval, and beta blocker medication, TWH-SD and QT-SD retained their significant power in discriminating between the victims of SCD and the patients with CAD (*p* = 0.00003, *p* = 0.006, respectively). TWH-SD outperformed QT-SD in identifying the SCD victims among the study subjects (area under the curve in the receiver operating characteristics curve 0.730 vs. 0.679, respectively).

**Conclusion:**

Increased short-term variability of repolarization heterogeneity measured from standard 12-lead ECG is associated with SCD.

## Introduction

Temporal variability of electrocardiographic repolarization has usually been evaluated from long-term electrocardiographic recordings rather than from a standard 12-lead electrocardiogram (ECG). Earlier studies have used several different approaches, such as the evaluation of QT/RR relationship, circadian pattern of heart rate corrected QT interval (QTc), various methods describing temporal variability of QT interval, and T-wave alternans (TWA). The slope of QT/RR relationship over 24 h has been found to be significantly higher in post-infarction patients with malignant ventricular tachyarrhythmias (Kluge et al., 1997). Post-infarction patients with life-threatening arrhythmias have been observed to have more commonly QTc peaks longer than 500 ms on Holter recordings (Homs et al., 1997). One of the parameters describing temporal variability of QT interval is QT variability index (QTVI) (Berger et al., 1997). It has been found to be associated with an increased risk for ventricular tachycardia or ventricular fibrillation in post-infarction patients with severely decreased left ventricular function (Haigney et al., 2004). Ambulatory ECG-based TWA has been shown to predict fatal cardiac events including sudden cardiac death (SCD) (Quan et al., 2014). Increased spatial heterogeneity of repolarization measured from standard 12-lead ECG has been associated with the risk of SCD (Porthan et al., 2013). Waks et al. (2015) evaluated the mean angle between consecutive T-wave vectors on standard 12-lead ECG and found that it was associated with SCD in community-based cohort of participants. However, data on the significance of temporal variability of spatial heterogeneity of repolarization analyzed from standard 12-lead ECG are limited. Therefore, in the present case–control study, we tested the hypothesis that beat-to-beat variability of parameters describing spatial heterogeneity of repolarization determined from standard 12-lead ECG discriminate the victims of unexpected SCD verified to be due to atherosclerotic coronary artery disease (CAD) by autopsy from the CAD patients who remained alive during the follow-up.

## Materials and Methods

### Study Populations

For the present case–control study, the cases of the victims of unexpected SCD determined to be due to atherosclerotic coronary disease by autopsy were drawn from the Fingesture (Finnish Genetic Study of Arrhythmic Events) database (Kaikkonen et al., 2006; Haukilahti et al., 2019). The age- and sex-matched control patients with angiographically verified CAD were drawn from the ARTEMIS (The Innovation to Reduce Cardiovascular Complications of Diabetes at the Intersection; NCT01426685) database (Junttila et al., 2018).

In collaboration with the Finnish Institute for Health and Welfare, the Department of Forensic Medicine and Cardiology Group at the University of Oulu have assembled the Fingesture database, which contains the medico-legal autopsy data from 5869 victims of SCD (Kaikkonen et al., 2006; Haukilahti et al., 2019). All of the study subjects have died due to SCD between 1998 and 2017. The medico-legal autopsies were done in the Forensic Medicine Unit of the Finnish Institute for Health and Welfare, Oulu, Finland, and at the Department of Forensic Medicine, University of Oulu, Oulu, Finland. The purpose of the systemically collected Fingesture database is to study both autopsy and clinical data of SCD subjects in Northern Finland. The whole study population is from a defined geographical area in Northern Finland. According to Finnish law, medico-legal autopsy is mandatory, if the death does not occur due to known preexisting medical condition, if the patient has not been treated by physicians during the last diseases, or if the death is unexpected (Act on the Inquest Into the Cause of Death, 459/1973, 7th paragraph: Finnish law). Adept forensic pathologists carried out all autopsies utilizing contemporary guidelines for diagnosis of cause of death. Death was classified as sudden if it was either witnessed within 6 h of the onset of symptoms or unwitnessed within 24 h when the subject was last seen alive in a normal state of health. Non-cardiac causes of sudden death were excluded from the study. SCD was further classified into ischemic and non-ischemic causes. The criteria for ischemic SCD included evidence of an active coronary artery process defined as an acute intracoronary thrombus, plaque rupture or erosion, hemorrhage into a plaque or critical coronary stenosis (>75%) in major coronary artery (Kaikkonen et al., 2006; Haukilahti et al., 2019). The study was approved by the Ethics Committee of Northern Ostrobothnia Hospital District, and the study complies with the Declaration of Helsinki. Permits to use data from medico-legal death investigations were obtained from the Finnish Institute for Health and Welfare and the Regional State Administrative Agency of Northern Finland. The part of the database, which was respectively analyzed during this study was assembled between 2013 and 2017. Database includes the last ECGs of the adult patients in digital format (*n* = 259). Subjects with non-sinus rhythm or unanalyzable recording were excluded from this study. After the exclusions, a total of 200 analyzable recordings remained in the study.

Subsequently, 200 age- and sex-matched controls were drawn from the ARTEMIS database, which consists of 1946 patients with CAD with angiographically confirmed stenosis of ≥50% in at least one major coronary artery. The control patients were event-free during the study follow-up (73 ± 22 months) and all subjects were in sinus rhythm. The study complies with the Declaration of Helsinki, and it was reviewed and approved by the Ethics Committee of Northern Ostrobothnia Hospital District. All subjects included in the study provided an informed consent. The details of the ARTEMIS study are described elsewhere (Junttila et al., 2018).

### Electrocardiography

**FIGURE 1:**
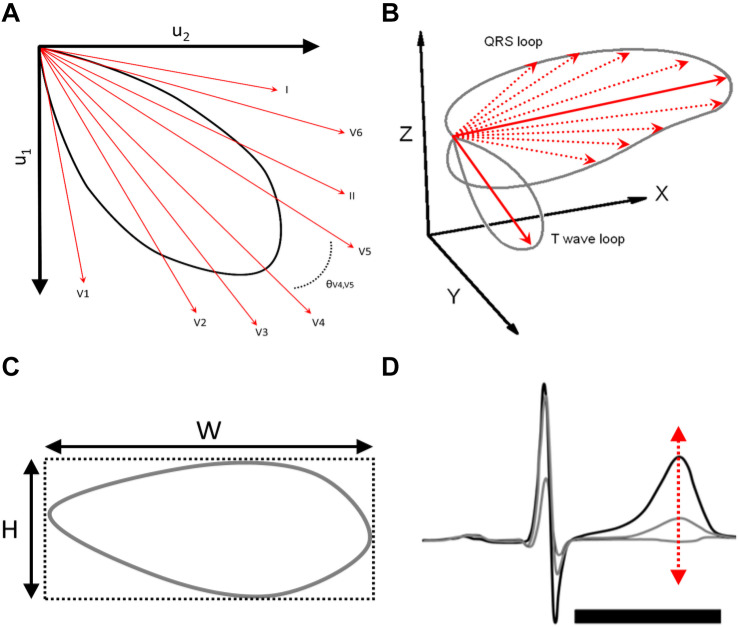
**(A)** T-wave morphology dispersion (degrees) (TMD) is based on the T-wave loop. TMD is obtained by calculating the average angle between all possible reconstruction vector pairs of limb leads I–II and chest leads V2–V6. **(B)** Total cosine R-to-T reflects the spatial angle between main QRS vector and the main T-wave vector (TCRT). **(C)** T-window-H was defined as the height of the window encompassing T-wave loop (mV), and T-window-W was defined as the width of the window encompassing T-wave loop (mV). **(D)** T-wave heterogeneity (TWH) was obtained from left precordial leads (V4–V6) using second central moment analysis. It is a measure of splay of waveforms around the average waveform of T-wave.

The standard 12-lead ECG recordings were assembled individually from each SCD victim’s health records. The mean duration from 12-lead ECG recording to SCD was 1.8 ± 2.6 months. Each ECG of the victims of SCD and the patients with CAD was analyzed in digital form with custom-made software written in Matlab (MathWorks, Natick, MA). Repolarization metrics were computed automatically. QT interval (QT) was measured from the beginning of the QRS complex to the end of T-wave. The 12-lead ECG signal was decomposed to eight singular values in a subspace in which the first three components contain most of the ECG energy. These three components were used to reconstruct T-waves and QRS complexes and determine their corresponding loops (Acar et al., 1999; Zabel et al., 2000, 2002). T-window-H was defined as the height of the window encompassing T-wave loop (mV), and T-window-W was defined as the width of the window encompassing T-wave loop (mV). Total cosine R-to-T reflects the spatial angle between main QRS vector and the main T-wave vector (TCRT) (Zabel et al., 2000, 2002). T-wave morphology dispersion (degrees) (TMD) is based on the T-wave loop. It is a measure of repolarization heterogeneity. TMD is obtained by calculating the average angle between all possible reconstruction vector pairs of limb leads I–II and chest leads V2–V6 (Porthan et al., 2013). Similar morphologies between these leads result in small TMD values indicative of low T-wave heterogeneity, whereas dissimilarities between the leads indicate increased spatial heterogeneity and result in greater TMD values. Normally, the reconstruction vector of V1 is far from the others and is excluded from the analysis (Acar et al., 1999). TMDpre was defined as TMD for ascending part of the T-wave and TMDpost as TMD for descending part of the T-wave. T-wave heterogeneity (TWH) was obtained from left precordial leads (V4–V6) using second central moment analysis. It is a measure of splay of waveforms around the average waveform of T-wave (Nearing and Verrier, 2003; Kenttä et al., 2016). TMD, TCRT, T-window-H, and T-window-W as well as TWH are illustrated in Figure 1. The beat-to-beat variability of repolarization parameters was analyzed from five consecutive sinus beats from standard 12-lead ECG by calculating standard deviation (SD) of the parameter within the segment. All subjects were in sinus rhythm, and no premature ventricular contractions were included in the measured segment.

### Statistical Analysis

The statistical significance of differences between continuous variables was assessed using the standard *t*-test and between categorical variables using the Chi-square test. When comparing repolarization parameters, corrections for multiple comparisons were made by multiplying the significant *p*-values by 16 (number of comparisons). The multivariate logistic regression analysis and the Cox multivariate hazards model were used to assess the independent value of the variables in discriminating between the victims of SCD and the patients with CAD. The receiver operating characteristics (ROC) curves were computed for TWH-SD, QT-SD, and TWH to show accuracies of these parameters in identifying the victims of SCD. A *p*-value < 0.05 was considered as statistically significant.

## Results

The clinical characteristics of the study subjects are shown in Table 1. The patients with CAD more commonly had diabetes and hypertension; were more often on beta blocker, statin, aspirin, angiotensin-converting-enzyme inhibitor/angiotensin II receptor blocker, and calcium channel blocker medication; and had longer RR interval (RR) but similar RR-SD compared with the victims of SCD.

**TABLE 1:** Clinical characteristics of study subjects.

Variable	Patients with CAD (*n* = 200)	Victims of SCD (*n* = 200)	*p*-value
Age, years	65 ± 10	64 ± 11	0.36
Sex, male	70.5%	70.5%	1.00
Diabetes	44%	22.5%	<0.001
Hypertension	64%	36%	<0.001
LBBB	0%	0%	1.00
RBBB	0%	1.5%	0.08
RR, ms	1026 ± 168	853 ± 181	<0.001
RR-SD, ms	21.4 ± 27.4	18.3 ± 26.7	0.25
β-Blocker	89%	20.5%	<0.001
Statin	92%	11%	<0.001
Aspirin	97%	14%	<0.001
Diuretics	29.5%	16%	0.09
ACEI/ARB	67.5%	15%	<0.001
Ca-blocker	24.5%	5.5%	<0.001

*The values are mean ± SD or percentages. ACEI/ARB, angiotensin-converting-enzyme inhibitor/angiotensin II receptor blocker; Ca-blocker, calcium channel blocker; CAD, coronary artery disease; LBBB, left bundle branch block; RBBB, right bundle branch block; RR, RR interval; SCD, sudden cardiac death; SD, standard deviation. See “Materials and Methods” section for details.*

The victims of SCD had longer QTc interval compared with the patients with CAD. Of the electrocardiographic parameters describing spatial heterogeneity of repolarization, the values of T-window-H and TWH were significantly higher and the values of TCRT were significantly lower in the victims of SCD than in the patients with CAD (Table 2).

**TABLE 2:** Repolarization parameters and their temporal variability.

Variable	Patients with CAD (*n* = 200)	Victims of SCD (*n* = 200)	*p*-value
QTc, ms	397 ± 26	416 ± 38	5.0E-9 (0.00000008)
QT-SD, ms	3.8 ± 7.1	8.3 ± 13.1	0.00003 (0.00048)
T-window-H, mV	0.095 ± 0.053	0.107 ± 0.061	0.027 (0.43)
T-window-H-SD, mV	0.0068 ± 0.0046	0.0094 ± 0.0057	5.9E-7 (0.0000094)
T-window-W, mV	0.32 ± 0.14	0.33 ± 0.16	0.68
T-window-W-SD, mV	0.012 ± 0.007	0.014 ± 0.014	0.24
TCRT	0.21 ± 0.54	0.10 ± 0.53	0.04 (0.64)
TCRT-SD	0.058 ± 0.053	0.088 ± 0.126	0.002 (0.032)
TMD, degrees	33.2 ± 24.8	33.8 ± 25.5	0.79
TMD-SD, degrees	2.41 ± 2.30	3.24 ± 3.79	0.009 (0.14)
TMDpre, degrees	35.0 ± 24.8	36.0 ± 26.2	0.69
TMDpre-SD, degrees	2.79 ± 2.58	4.66 ± 5.20	0.000007 (0.000112)
TMDpost, degrees	26.0 ± 31.2	25.9 ± 29.0	0.96
TMDpost-SD, degrees	2.58 ± 3.80	3.84 ± 5.94	0.012 (0.19)
TWH, μV	58.7 ± 40.4	83.0 ± 57.1	0.000001 (0.000016)
TWH-SD, μV	3.8 ± 2.6	6.9 ± 5.6	1.8E-11 (0.000000000288)

*The values are means ± SD. CAD, coronary artery disease; QT, QT interval; QTc, QT interval corrected for heart rate using the Bazett’s formula; SCD, sudden cardiac death; SD, standard deviation; TCRT, total cosine R-to-T; reflects the spatial angle between main QRS vector and the main T-wave vector; TMD, T-wave morphology dispersion (degrees); TMDpost, TMD for descending part of the T wave (degrees); TMDpre, TMD for ascending part of the T-wave (degrees); T-window-H, height of the window encompassing T-wave loop (mV); T-window-W, width of the window encompassing T-wave loop (mV); TWH, T-wave heterogeneity in leads V4–V6. The p-values shown in parentheses are adjusted for multiple comparisons by multiplying the significant p-values by 16 (number of comparisons). See “Materials and Methods” section for details.*

Temporal variability of repolarization analyzed from five consecutive beats of the standard 12-lead ECG represented by QT-SD, T-window-H-SD, TCRT-SD, TMD-SD, TMDpre-SD, TMDpost-SD, and TWH-SD was significantly higher in the victims of SCD than in the subjects with CAD. The *p*-values shown in parentheses are adjusted for multiple comparisons by multiplying the significant *p*-values by 16 (number of comparisons). As can be seen, statistically significant *p*-values for important comparisons were still held (Table 2). When analyses were performed in males and females separately, the results were similar (data not shown).

When the clinical variables including beta blocker medication that differed significantly between the subjects with CAD and the victims of SCD (Table 1) were tested together in the multivariate logistic regression analysis, diabetes, RR interval, and beta blocker medication remained in the model as significant factors that discriminated the study groups. When the repolarization parameters that differed significantly between the patients with CAD and the victims of SCD (Table 2) were tested one at a time in this clinical model, QT-SD, TCRT-SD, TWH-SD, and TWH retained their significant discriminating power between the study groups after adjustments (Table 3). When the relevant clinical variables that differed significantly between the subjects with CAD and the victims of SCD (Table 1) were tested together in the Cox multivariate regression analysis, RR interval and beta blocker medication retained their significant discriminating power. When the repolarization parameters that differed significantly between the patients with CAD and the victims of SCD (Table 2) were tested one at a time in this clinical model, QT-SD, TCRT-SD, and TWH-SD retained their significant discriminating power between the study groups after adjustments. TWH and TMDpost-SD had *p*-values of borderline significance after adjustments in the Cox multivariate hazards model (Table 4). The time from the 12-lead ECG recording to SCD in SCD victims and to the end of follow-up in CAD patients was used as a time factor in the Cox regression analyses.

**TABLE 3:** Association of repolarization parameters with risk of sudden cardiac death in multivariate analysis.

Variable	Relative risk	95% CI	*p*-value
QTc, ms	1.004	0.994–1.015	0.43
QT-SD, ms	1.047	1.013–1.082	0.006
T-window-H, mV × 10^–2^	1.043	0.988–1.10	0.13
T-window-H-SD, mV × 10^–3^	1.05	0.983–1.121	0.14
TCRT × 10^–1^	1.00	0.947–1.057	0.99
TCRT-SD × 10^–2^	1.044	1.003–1.085	0.03
TMD-SD, degrees	1.036	0.941–1.139	0.47
TMDpre-SD, degrees	1.057	0.968–1.153	0.22
TMDpost-SD, degrees	1.05	0.992–1.112	0.09
TWH, μV	1.014	1.007–1.021	0.0002
TWH-SD, μV	1.255	1.127–1.398	0.00003

*CI, confidence interval. The other abbreviations are the same as in Table 2. When the clinical variables including beta blocker medication that differed significantly between the subjects with CAD and the victims of SCD (Table 1) were tested together in the multivariate logistic regression analysis, diabetes, RR interval, and beta blocker medication remained in the model as significant factors that discriminated the study groups. The repolarization parameters that differed significantly between the patients with CAD and the victims of SCD (Table 2) were tested one at a time in this clinical model.*

**TABLE 4:** Association of repolarization parameters with risk of sudden cardiac death in cox multivariate regression analysis.

Variable		HR	95% CI	*p*-value
QTc, ms	uv	1.012	1.008–1.016	3.1E-9
	mv	1.002	0.997–1.007	0.46
QT-SD, ms	uv	1.021	1.012–1.031	0.000013
	mv	1.016	1.002–1.031	0.024
T-window-H, mV × 10^–2^	uv	1.024	1.001–1.047	0.043
	mv	1.021	0.994–1.047	0.12
T-window-H-SD, mV × 10^–3^	uv	1.053	1.032–1.075	5.9E-7
	mv	1.016	0.990–1.042	0.24
TCRT × 10^–1^	uv	0.972	0.948–0.997	0.028
	mv	0.987	0.960–1.016	0.38
TCRT-SD × 10^–2^	uv	1.019	1.008–1.031	0.001
	mv	1.012	1.000–1.025	0.047
TMD-SD, degrees	uv	1.054	1.018–1.092	0.003
	mv	1.028	0.981–1.078	0.25
TMDpre-SD, degrees	uv	1.061	1.036–1.087	0.000001
	mv	1.029	0.995–1.064	0.096
TMDpost-SD, degrees	uv	1.035	1.011–1.059	0.004
	mv	1.026	1.000–1.052	0.054
TWH, μV	uv	1.005	1.003–1.007	0.000007
	mv	1.002	1.000–1.005	0.062
TWH-SD, μV	uv	1.070	1.051–1.090	5.3E-13
	mv	1.054	1.020–1.090	0.002

*CI, confidence interval; HR, hazards ratio; mv, multivariate; uv, univariate. The other abbreviations are the same as in Table 2. When the relevant clinical variables that differed significantly between the subjects with CAD and the victims of SCD (Table 1) were tested together in the Cox multivariate regression analysis, RR interval and beta blocker medication retained their significant discriminating power. The repolarization parameters that differed significantly between the patients with CAD and the victims of SCD (Table 2) were tested one at a time in this clinical model. The time from the 12-lead ECG recording to SCD in SCD victims and to the end of follow-up in CAD patients was used as a time factor in the Cox regression analyses.*

**FIGURE 2:**
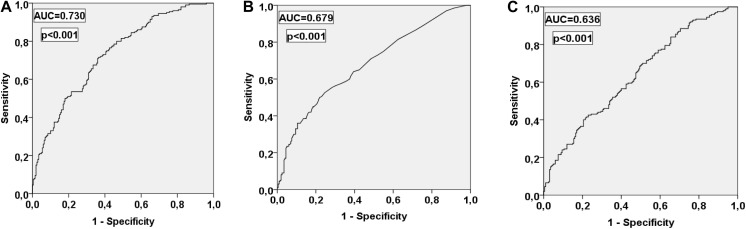
Receiver operating characteristic curves for TWH-SD **(A)**, QT-SD **(B)**, and TWH **(C)** in discriminating the victims of SCD from the patients with CAD. AUC, area under the curve. Other abbreviations are the same as in Table 2.

TWH-SD, QT-SD, and TWH were the repolarization parameters, which had the strongest discriminating power between the patients with CAD and the victims of SCD in the multivariate analysis (Table 3). The ROC curves show the accuracies of these parameters in identifying the SCD victims among the study subjects, with TWH-SD outperforming TWH and QT-SD (Figure 2). At the cutoff point of 5.22 optimized from the ROC curve, TWH-SD had 54% sensitivity, 79% specificity, 71% positive predictive value, and 63% negative predictive value in discriminating SCD victims from control patients. At the cutoff point of 3.52, the corresponding values for QT-SD were 50%, 78%, 69%, and 61%, and at the cutoff point of 79.51, the corresponding values for TWH were 43%, 76%, 64%, and 57%, respectively.

Beta blocker medication did not have significant association with TWH-SD, TWH, or QT-SD in the patients with CAD or in the victims of SCD (Table 5). RR interval had significant but relatively weak positive correlation with TWH in the patients with CAD, suggesting a weak association of higher TWH values with lower heart rate in the patients with CAD of the present study. RR interval did not have any other significant associations with TWH-SD, TWH, or QT-SD either in the patients with CAD or the victims of SCD (Table 6).

**TABLE 5:** Association of beta blocker medication with TWH-SD, TWH, and QT-SD.

(A) In patients with CAD			

Variable	No beta blocker medication	Beta blocker medication	*p*-value
TWH-SD	3.9 ± 2.8, μV	3.8 ± 2.6, μV	0.88
TWH	60.4 ± 37.5, μV	58.5 ± 40.9, μV	0.83
QT-SD	2.9 ± 2.9, ms	3.9 ± 7.4, ms	0.51

**(B) In victims of SCD**			

**Variable**	**No beta blocker medication**	**Beta blocker medication**	***p*-value**

TWH-SD	6.6 ± 4.0, μV	6.3 ± 5.9, μV	0.79
TWH	84.4 ± 59.3, μV	78.3 ± 54.5, μV	0.57
QT-SD	7.1 ± 10.2, ms	8.5 ± 13.0, ms	0.50

*The values are means ± SD. The abbreviations are the same as in Table 2.*

**TABLE 6:** Association of RR Interval with TWH-SD, TWH, and QT-SD.

(A) In patients with CAD	
**Variable**	**RR Interval**

TWH-SD	0.11 (0.12)
TWH	0.21 (0.002)
QT-SD	−0.01 (0.88)

**(B) In victims of SCD**	

**Variable**	**RR Interval**

TWH-SD	−0.13 (0.08)
TWH	0.12 (0.10)
QT-SD	−0.12 (0.08)

*The values are the Spearman correlation coefficients (p-values in parentheses). The abbreviations are the same as in Table 2.*

## Discussion

In the present study, we found that temporal variability of spatial heterogeneity of repolarization, TWH-SD in particular, analyzed from five consecutive beats of standard 12-lead ECG identified the SCD victims with relatively good accuracy. Temporal variability of QT interval and spatial heterogeneity of repolarization described by TWH also discriminated the SCD victims from the control subjects with CAD. However, temporal variability of TWH outperformed QT-SD and TWH in identifying the victims of SCD.

QTc, an electrocardiographic measure of repolarization duration, has been shown to predict SCD in post-infarction patients (Schwartz and Wolf, 1978) and to also yield prognostic information in healthy subjects (Schouten et al., 1991). In the past decades, there has been a growing interest in newer parameters that are based on T-wave loop analysis and describe spatial heterogeneity of repolarization (Zabel et al., 2000, 2002). T-wave morphology parameters, such as TMD, have been shown to predict SCD in the general population (Porthan et al., 2013). In a study including patients with CAD, T-wave morphology parameters, such as TMD, T-wave area dispersion, and TCRT, were associated with cardiac death (Pirkola et al., 2018). Spatial heterogeneity of repolarization evaluated from standard 12-lead ECG from left precordial leads (V4–V6) by TWH using second central moment analysis has been shown to predict SCD in the general population (Kenttä et al., 2016). In alignment with this observation, TWH identified the SCD victims in our present study even after relevant adjustments in the multivariate logistic regression analysis. Several other parameters describing repolarization duration or spatial heterogeneity of repolarization, such as QTc, T-window-H, and TCRT, were associated with the risk of SCD, but only TWH retained its discriminating power between the SCD victims and CAD patients after relevant adjustments. Concurring with these notions, there are previous data to show that lateral ST depression with or without T-wave changes on standard 12-lead ECG is an independent predictor of cardiac death after myocardial infarction (Perkiomaki et al., 2002a).

However, temporal variability of TWH was more closely associated with the risk of SCD than TWH that describes merely spatial heterogeneity of repolarization and not beat-to-beat changes of spatial heterogeneity of repolarization. TWH-SD clearly outperformed TWH in discriminating between the SCD victims and CAD patients. This notion suggests that the evaluation of temporal variability of spatial heterogeneity of repolarization yields incremental prognostic information over the evaluation of spatial heterogeneity of repolarization. One potential mechanism why increased spatial and temporal variability of repolarization increases the risk for SCD is that they favor re-entry, which may initiate life-threatening ventricular tachyarrhythmias.

Previous studies have mainly evaluated temporal changes of repolarization from electrocardiographic recordings by assessing the variability of repolarization duration using QT interval or its subintervals in several beat blocks or on a beat-to-beat basis. The evaluation of the QT/RR slope has been shown to yield important information about the alterations in repolarization dynamics in various pathological conditions. Post-infarction patients with malignant ventricular tachyarrhythmias have been found to have higher slope of QT/RR relationship over 24 h than post-infarction patients without such arrhythmias or healthy subjects (Kluge et al., 1997). Studies on circadian patterns of QTc have observed higher proportion of QTc peaks longer than 500 ms in post-infarction patients with life-threatening arrhythmias (Homs et al., 1997). QTVI, which is the log ratio between the QT interval variability and heart rate variability, each normalized by the squared mean of the respective time series was developed in 1997 (Berger et al., 1997). It has been shown to identify patients with SCD in a mixed patient population referred for electrophysiological studies (Atiga et al., 1998) and to predict ventricular tachycardia or ventricular fibrillation determined by implantable cardioverter–defibrillator interrogation in post-infarction patients with severely decreased left ventricular function (Haigney et al., 2004). Generally, the higher the temporal variability of repolarization and the lower the heart rate variability, the higher the risk. It has been suggested that autonomic nervous influences on ventricular repolarization and heart rate are qualitatively similar in normal individuals (Merri et al., 1993) but may be dissimilar in pathological conditions (Merri et al., 1992). Increased temporal complexity of the interval between the R-peak and the apex of the T-wave has been observed to predict mortality in high-risk patients with implantable cardioverter–defibrillators and left ventricular dysfunction (Perkiomaki et al., 2003). QT variability and the variability of spatial T-wave complexity have been found to be increased in patients with the congenital long QT syndrome despite unchanged heart rate variability, suggesting that there is divergence of repolarization and heart rate dynamics possibly increasing vulnerability to arrhythmias (Perkiomaki et al., 2002b). Analogously in the present study, even though the temporal variability of repolarization was increased, heart rate variability was similar, in the SCD victims compared with the patients with CAD. Periodic repolarization dynamics, a novel marker of sympathetic activity, has been shown to predict SCD and non-SCD in post-infarction patients with decreased left ventricular function (Rizas et al., 2017).

If the prognostic information included in the temporal variability of electrocardiographic spatial heterogeneity of repolarization could be obtained from the standard 12-lead ECG, that would open better opportunities for possible clinical applications. The work by Waks et al. (2015) is one of the few studies that have evaluated the prognostic significance of temporal dynamics of repolarization from the standard 12-lead ECG. They found that the mean angle between consecutive T-wave vectors on standard 12-lead ECG was associated with SCD in the general population. Our present findings show that TWH-SD, which was analyzed from five consecutive beats of the standard 12-lead ECG and describes temporal variability of spatial heterogeneity of repolarization, discriminates the victims of SCD from the patients with CAD relatively well. TWH-SD outperformed TWH, which is a measure of spatial heterogeneity of repolarization and QT-SD that describes temporal variability of repolarization duration, in identifying the SCD victims. Our observations support the concept that the prognostic value of spatial heterogeneity of repolarization is improved by evaluating the temporal variability of spatial heterogeneity of repolarization.

One of the strengths of our study is that the causes of sudden death were confirmed to be cardiac in medico-legal autopsy. The victims of SCD were drawn from the Fingesture database (Kaikkonen et al., 2006; Haukilahti et al., 2019), which is the largest consecutive series of medico-legal autopsy verified SCD victims in the world. However, the study also has some limitations. There were some differences in clinical characteristics between the study groups. However, the study groups were matched according to age and sex, and the main results remained significant after relevant adjustments in the multivariate model. In addition, beta blocker medication did not have any significant association with TWH-SD, TWH, or QT-SD either in the patients with CAD or in the victims of SCD. Furthermore, no significant confounding associations were observed between these parameters and RR interval in either of the study groups, and RR interval variability was similar between the groups.

## Conclusion

In conclusion, temporal variability of spatial heterogeneity of repolarization analyzed from the standard 12-lead ECG was a better discriminator than spatial heterogeneity of repolarization between the victims of unexpected SCD determined to be due to complicated atherosclerotic coronary disease by autopsy and the patients with angiographically verified CAD. Our findings should be confirmed in follow-up studies with relevant endpoints including SCD.

## Data Availability Statement

The raw data supporting the conclusions of this article will be made available by the authors, without undue reservation, to any qualified researcher.

## Ethics Statement

The studies involving human participants were reviewed and approved by the Ethics Committee of the Northern Ostrobothnia Hospital District, and the study complies with the Declaration of Helsinki. The patients/participants provided their written informed consent to participate in this study.

## Author Contributions

JH participated in the design of the study, matching the study groups, analyzed the study parameters, did the initial statistical analyses, and wrote the first version of the manuscript. TK participated in the design of the study, matching the study groups, analyses of the study parameters, and drafting the manuscript. MH, JR, LH, JV, MT, AK, LP, OU, MJ, and HH participated in the collection and analyses of the data of the study populations. JP participated in the design of the study, collection of the data for study groups, the statistical analyses, and wrote the final version of the manuscript. All authors contributed to the article and approved the submitted version.

## Conflict of Interest

The authors declare that the research was conducted in the absence of any commercial or financial relationships that could be construed as a potential conflict of interest.
